# Identifying and prioritizing strategies for comprehensive liver cancer control in Asia

**DOI:** 10.1186/1472-6963-11-298

**Published:** 2011-11-02

**Authors:** John FP Bridges, Gisselle Gallego, Masatoshi Kudo, Kiwamu Okita, Kwang-Hyub Han, Sheng-Long Ye, Barri M Blauvelt

**Affiliations:** 1 Department of Health Policy and Management Johns Hopkins Bloomberg School of Public Health 624 N. Broadway, Room 689 Baltimore, MD 212105 USA; 2 Department of Gastroenterology and Hepatology Kinki University School of Medicine 377-2 Ohno-Higashi, Osaka-Sayama Osaka, Japan; 3 Yamaguchi University Shimonoseki Kohsei Hospital Kamishinchi-cho 3-3-8 Shimonoseki City, Japan; 4 Division of Gastroenterology Department of Internal Medicine Chief, Liver Cancer Special Clinic Severance Hospital Director, Liver Cirrhosis Clinical Research Center Yonsei University College of Medicine 134 Shinchon-dong, Seodaemun-gu Seoul, Korea; 5 Liver Cancer Institute Zhongshan Hospital Fudan University 136 Yixueyuan Road Shanghai, PR China; 6 Institute for Global Health, University of Massachusetts, 102 Hasbrouck, University of Massachusetts Amherst, MA 01035, USA

## Abstract

**Background:**

Liver cancer is both common and burdensome in Asia. Effective liver cancer control, however, is hindered by a complex etiology and a lack of coordination across clinical disciplines. We sought to identify strategies for inclusion in a comprehensive liver cancer control for Asia and to compare qualitative and quantitative methods for prioritization.

**Methods:**

Qualitative interviews (N = 20) with international liver cancer experts were used to identify strategies using Interpretative Phenomenological Analysis and to formulate an initial prioritization through frequency analysis. Conjoint analysis, a quantitative stated-preference method, was then applied among Asian liver cancer experts (N = 20) who completed 12 choice tasks that divided these strategies into two mutually exclusive and exhaustive subsets. Respondents' preferred plan was the primary outcome in a choice model, estimated using ordinary least squares (OLS) and logistic regression. Priorities were then compared using Spearman's Rho.

**Results:**

Eleven strategies were identified: *Access to treatments; Centers of excellence; Clinical education; Measuring social burden; Monitoring of at-risk populations; Multidisciplinary management; National guidelines; Public awareness; Research infrastructure; Risk-assessment and referral*; and *Transplantation infrastructure*. Qualitative frequency analysis indicated that *Risk-assessment and referral *(85%), *National guidelines *(80%) and *Monitoring of at-risk populations *(80%) received the highest priority, while conjoint analysis pointed to *Monitoring of at-risk populations *(p < 0.001), *Centers of excellence *(p = 0.002), and *Access to treatments *(p = 0.004) as priorities, while *Risk-assessment and referral *was the lowest priority (p = 0.645). We find moderate concordance between the qualitative and quantitative methods (rho = 0.20), albeit insignificant (p = 0.554), and a strong concordance between the OLS and logistic regressions (rho = 0.979; p < 0.0001).

**Conclusions:**

Identified strategies can be conceptualized as the ABCs of comprehensive liver cancer control as they focus on *Antecedents*, *Better care *and *Connections *within a national strategy. Some concordance was found between the qualitative and quantitative methods (e.g. *Monitoring of at-risk populations*), but substantial differences were also identified (e.g. qualitative methods gave highest priority to risk-assessment and referral, but it was the lowest for the quantitative methods), which may be attributed to differences between the methods and study populations, and potential framing effects in choice tasks. Continued research will provide more generalizable estimates of priorities and account for variation across stakeholders and countries.

## Background

Hepatocellular carcinoma (HCC), the predominant form of liver cancer, is the sixth most common cancer and the third most frequent cause of cancer-related death worldwide [1,2]. At least two thirds of the people who die each year from HCC live in the Asia-Pacific region [3]. The majority of patients with HCCs are diagnosed in the advanced stages of presentation due to the relative paucity of symptoms in the early stages [4]. Because of the multifocal and advanced stage of disease at time of diagnosis, potentially curative treatment for HCC is not feasible in 80% of patients [5].

Chronic liver disease is closely associated with HCC. In areas where hepatitis B virus (HBV) is endemic, the incidence of HCC is high. It has been estimated that about 75% of the world's chronic HBV carriers are in Asia [6]. However, the etiology of HCC in Japan is different as hepatitis C virus (HCV) is more prevalent than HBV. Ninety percent of the HCC in Japan is HCV related [5]. As stated in a recent report by the United States Institute of Medicine, both HBV and HCV can be prevented and controlled, which would reduce the incidence of HCC and liver disease [7].

The relative burden and complexity of liver cancer, especially in Asia, lends itself to a comprehensive cancer control plan. However, there is a paucity of data or experience to design such a policy response. While comprehensive cancer control plans regularly target lung, colorectal, breast and cervical cancer, such approaches have not been applied to liver cancer [8]. The WHO guidance for the development of national cancer programs offers some guidance for implementation [9]. The WHO conceptualizes its model around disease progression and is focused around six dimensions: prevention, early detection, diagnosis/treatment, pain relief/palliative care, cancer control research, and surveillance. One of the limitations of this approach is that it distinguishes between appropriate strategies that should be used in countries with low, middle and high levels of resources-a barrier to a common policy framework that would be appropriate for a pan-Asian response [10].

This paper reports the findings of a study aimed at identifying strategies appropriate for inclusion in a comprehensive liver cancer control plan and at assessing the relative priorities among these strategies. We also sought to compare the implied priorities in the qualitative data (i.e. via semi-quantification using frequency analysis) to those found using a quantitative stated-preference methodology (conjoint analysis)-with a particular focus on Asia.

Our research is of interest to those focused on liver cancer control, especially in Asia, for three reasons. First, beyond clinical guidelines, there is very little in the way of comparative research on liver cancer policy internationally. This paucity of data extends even to basic epidemiological data on HCC, which are fragmented and come from diverse populations, using different methodologies and from studies performed at different times [2]. Second, while Japan and Taiwan have demonstrated successful strategies to combat HCC, especially through HBV vaccination and control, there is an absence of models of best practice for comprehensive liver cancer control beyond HBV vaccination in most countries of Asia [3,11,12]. Third, there are few templates available for the development of comprehensive cancer control plans for liver cancer, and it is uncertain if general cancer-control frameworks, such as the one proposed by the WHO [9], are relevant for liver cancer control.

We also make an important methodological contribution that is relevant to a wider audience of policy makers and health services researchers. Specifically, we demonstrate that while qualitative methods are valuable in identifying strategies [13], semi-quantification methods such as frequency analysis [14] may be less desirable for prioritization [15]. We demonstrate this by comparing our frequency data with the results of a conjoint analysis-a qualitative stated-preference method [16] that is increasingly used to identify priorities for health care policy [17-19] and more broadly in health services research [20-22].

## Methods

The study utilized both qualitative and quantitative research methods. First, in-depth, open-ended interviews were used to identify possible strategies and to explore possible priorities using frequency analysis, a common semi-quantification method [14]. Qualitative methods are an important method for identifying complex issues in health care, including priority setting [23,24], and are an important way to include clinical stakeholders in decision making processes [25-27], including the study of cancer care and coordination [28-30]. Second, quantitative stated-preference methods were used to focus more on the priorities for Asia and to compare the implied priorities based on the qualitative data.

All participants were informed about the study and its potential risks and benefits. Participation in the study was voluntary and respondents were not reimbursed for participation. The study was deemed exempt from human subjects consideration from the Johns Hopkins University, Bloomberg School of Public Health Institutional Review Board (IRB). All respondents were guaranteed anonymity and confidentially.

### Identification (qualitative)

Respondents for the qualitative study were purposively sampled to constitute a geographically and professionally diverse sample of clinical experts in liver cancer and related disease [31]. Potential respondents were actively involved in clinical practice, academic medical centers and/or policy relating to the prevention, detection and/or management of liver cancer. Potential respondents were identified through published literature, medical societies and peer referral. Respondents were included if they were i) working in liver disease and liver cancer in their country; ii) involved in HCC clinical practice and policy; iii) active members in national and international liver associations and/or published extensively in peer review journals, and were excluded if they were not board certified or licensed to practice medicine in their countries and with at least three years of clinical experience or were unwilling or unable to complete the interview within the period required for completion of all interviews.

It is clear that our focus on clinical expertise is restrictive, but we wanted to ground our results in those who actually implement liver cancer control. This said, other stakeholders, including patients, family members, nursing staff or community leaders, may have given important insights. While this certainly is a limitation in our study, we aimed at assessing complex clinical issues, and as such needed experts who were experienced with discussing national liver cancer policies.

Information about the study and an invitation to participate was sent to respondents via mail or email in English, and in the respondents' native language where necessary. If no response was received within two weeks, follow-up included a second email and/or telephone call.

Open-ended qualitative interviews were conducted via face-to-face interviews or, in a limited number of instances, as telephone interviews. Multiple interviewers were used so as to accommodate multiple languages, with many of the interviews completed by the study leaders (JB, BB), an important triangulation method.

After respondents were informed about the study and consented to participate, they were asked about their country's "*strategies to promote liver cancer prevention, treatment and research*" and then "*the main gaps in public policy*." Finally, respondents were asked "*if you had an opportunity to develop a comprehensive liver cancer control strategy, what elements would it cover?*"

Interviews were recorded, transcribed (translated where necessary) and systematically analyzed in conjunction with any field notes. Respondents were allowed and encouraged to discuss other factors via open-ended questioning, but conversations were facilitated through the use of a comprehensive *aide memoire *based on previous research [10]. While saturation of themes was achieved after 16 interviews, we completed 20 interviews to facilitate semi-quantification.

Analysis was guided by Interpretive Phenomenological Analysis (IPA) [32] in order to capture respondents' experiences, perceptions, practices, and processes associated with liver cancer control. Data were initially reviewed and coded by two researchers, including one who participated in data collection and one who did not. To ensure reliability, coding was compared and discussed with senior study members (JB and BB), and a final selection and appropriate labeling of identified themes was determined.

Triangulation methods included the use of multiple interviewers and analysts, geographical and professional heterogeneity of respondents, the comparison of transcripts with field notes, and comparison of results to the published literature via a targeted literature review. Content experts (MK, KO, K-HH and S-LY) were consulted to ensure the validity of interpretation and to resolve any ambiguity in the data. After this, the two researchers reviewed the data to identify representative quotes and to ensure the reliability of the coding. Finally, to ensure that this manuscript reported all relevant information, we utilized the RATS guidelines [33,34].

### Prioritization (qualitative)

The use of numeration and/or semi-quantification in qualitative research remains controversial [35,36]. This said, such methods are frequently used in health care research [37-39], and are called for by the RATS guidelines [34]. Within the framework of IPA, numeration through an analysis of the frequency with which a theme is supported can be used as an indicator of its importance. As noted by Smith and colleagues [32]:

"...it makes sense to think of the frequency with which emergent themes appear as one (though not the only) indication of the relative importance and relevance..."

To examine the potential relative importance of the identified strategies, we examined the frequency with which these strategies (and any sub-ordinate concepts) were discussed [40]. Rather than examine the frequency within a respondent, we report the percentage of the respondents making any reference to each of the indentified themes.

### Prioritization (quantitative)

As a means of offering a more quantitative assessment of importance, we developed and implemented conjoint analysis to examine the importance that respondents placed on the identified strategies. While it would have been beneficial to draw such data from the same respondents who participated in the qualitative research, it was decided to recruit new respondents from a single geographic region.

Conjoint-analysis methods, and more specifically discrete choice experiments, are grounded in both mathematical psychology and economic theory [41,42]. They are based on the notion of the assessment of multiple stimuli (referred to as objects or attributes) that are combined to create vignettes or profiles that are presented to respondents in order to evoke an action, choice, or valuation [43]. While such methods are widely used in health care [20-22], they have more recently been applied to examine issues associated with liver cancer control [44-46].

Our approach to conjoint analysis is similar to that of Bridges et al. [19], where conjoint analysis cards are developed to present a number of attributes (or objects) that do not vary across levels. Hence, for any given scenario in a conjoint analysis task, the attribute is either turned on or off [47]. Rather than identifying the best object in each profile, we present competing plans that represent mutually exclusive and exhaustive subsets of the 11 attributes identified in the qualitative section.

**Figure 1:**
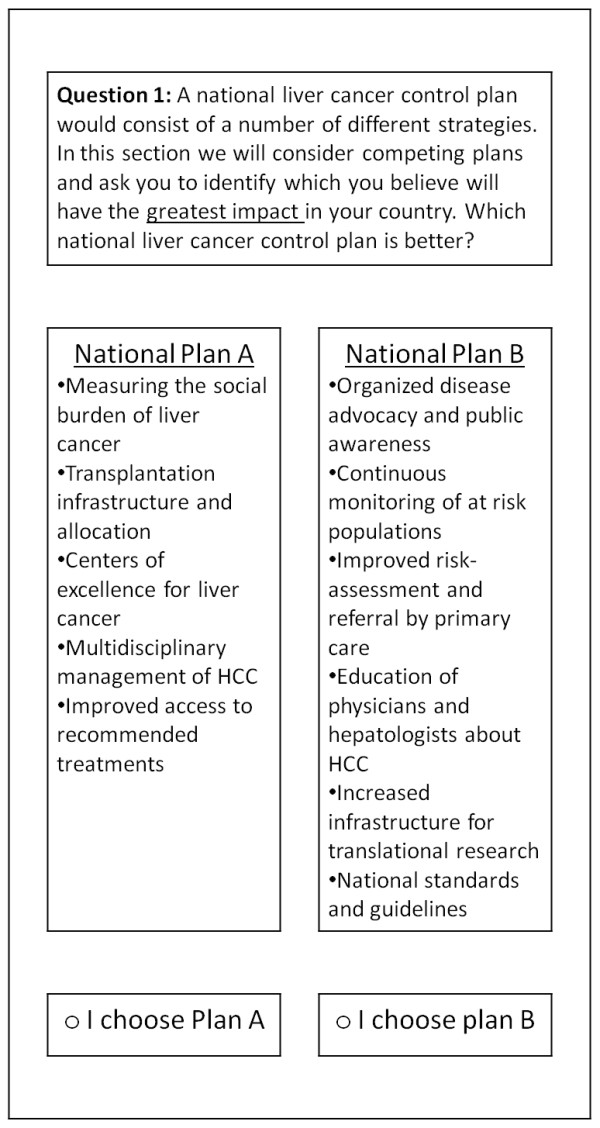
**An example of a conjoint analysis task**.

Our experimental design utilized a 2^11 main-effects orthogonal design from a catalogue of designs [48]. This design consisted of a 12 × 11 matrix, with each row representing a specific experiment and each column representing the 11 strategies identified from the qualitative method. Each cell in the matrix was either a 0 or 1 and in developing the pair tasks we interpreted 0 as implying that the strategy should be assigned to the left plan and 1 as assigning the strategy to the plan on the right. The properties of the design were rigorously tested and the results cards did constitute a balanced, orthogonal, and minimal (i.e. zero) overlap design [49]. An example of the conjoint analysis task is provided in Figure 1, where a respondent is asked to identify which of two national liver cancer control plans would have the most impact in their own country.

Potential respondents for the conjoint analysis were identified in China, Japan and South Korea by country experts (MK, KO, KH and SY) and the inclusion/exclusion criteria from our qualitative analysis were used, as were the recruitment procedures. Again, we did not recruit stakeholders other than clinicians, so our results may be biased towards their viewpoint. As the aim of this analysis was to compare the results of the frequency and conjoint analyses, we thought that it was appropriate to use a similar sample size (n = 20). While this is small for a conjoint analysis, it is similar to mixed methods preference studies found in the literature [13], and many commercial and legal applications of conjoint analysis methods have used similar sample sizes (especially when the focus is on the preferences of experts). Given this sample size, the results should not be interpreted as being widely generalizable, but comparable in scope to the qualitative research.

The quantitative survey instrument was administered to the respondents through a face-to-face interview or, where this was not possible, the survey was sent to the respondent and administered via a telephone interview. Respondents were guided through the questionnaire and answered the questions in the presence of the researcher. Respondents were asked to select the set of strategies they thought would be most important in a liver cancer control plan. No other answers or justifications were sought, and this process was repeated 12 times per participant. While some applications of conjoint analysis follow each task with an open or closed question regarding either strength of preference, ease of task or confidence in the answer [43], we did not include such questions so as to minimize the time burden on respondents. This said, notes were taken if respondents made any comments on the conjoint tasks.

The primary outcome in the analysis was the liver cancer control plan selected by the participant for each task, which was coded as a zero if the left-hand-side was chosen and one if the plan on the right was chosen. An identical method was used to code the placement of the strategies on the left and right of the choice tasks. For the purposes of comparison, we utilized both a linear probability model (via ordinary least squares) and logistic regression to estimate choice models using SAS (Version 9.13, Cary, NC, USA), but substantive conclusions are draw from the latter. For both estimation methods, robust standard errors are estimated to account for clustering of multiple choice tasks within each respondent [50,51]. Hypothesis testing was based on the null that respondents' choices were not affected by each strategy (i.e. the importance weight is zero). The natural alternative hypothesis was that the importance weights were positive (given that all factors were identified as having priorities), however, we allowed for strategies to have a negative sign (as was found in some previous research [52]), and utilized a two-tailed test.

To compare the implied priorities drawn from the qualitative and quantitative analyses, the estimated rank of the eleven strategies is presented graphically and in the results table. Further, the prioritization is compared between the qualitative and quantitative methods (and among the two quantitative estimation techniques) using the Spearman's Rho [53].

### Strengths and weaknesses

This is the first paper to focus on the development of strategies for inclusion in a comprehensive liver cancer control program, and in doing so we demonstrate three important issues. First, there is a paucity of robust scientific research to inform the development of evidence-based cancer control plans. Second, preferences-based methods, both qualitative and quantitative, are valuable in identifying and prioritizing control strategies from the perspective of local stakeholders. Finally, such methods offer an important alternative to consensus methods that can be driven by "strong personalities", rather than generalizable data.

There are also several weaknesses in the research underpinning this paper. First, while it is clear that this is subjective research, it is somewhat unclear who the best subjects to recruit are. In some respects our respondents are too homogeneous (i.e. clinicians with a national or international profile), and we have omitted many important viewpoints (other clinical experts, policy makers/leaders and patients/advocates). On the other side, our respondents are heterogeneous, spanning many countries that may have different priorities, which may be biasing our results towards the null. Second, while this method is focused on the comparison of two methods (one qualitative, one quantitative), they have different samples-the former being more international to identify a range of possible strategies, the latter focusing on only three, albeit geographically close, countries. Finally, we have used a rather small sample size to illustrate conjoint analysis, and a much larger sample would be required to ensure generalizability of our results.

Given these weaknesses, there are several limitations in our research that must be addressed. First, while the study was primarily focused on the identification of possible strategies, this should not be considered as an exhaustive set. Second, while this study presents data on priorities, the primary purpose is to demonstrate the limitation of qualitative methods in identifying priorities and to illustrate the benefit of conjoint analysis, not to offer a definitive prioritization of strategies for Asia. Finally, although the data presented here are somewhat novel, more research is needed to see how priorities vary across countries and other stakeholders and to identify which priorities are common in Asia, and which are specific to individual countries in the region.

## Results

### Identification (qualitative)

Invitations to participate in the open-ended interviews were sent to 25 possible respondents, all of who met the eligibility criteria. One respondent refused to participate, and a further four consented, but a mutually agreeable time to schedule the interview could not be identified before the desired number of respondents was reached. Twenty interviews were conducted between February and June 2010 with experts based in eleven different countries (Australia, China, France, Germany, Italy, Japan, Spain, South Korea, Taiwan, Turkey and United States). The average duration of the interviews was 34 minutes (range 16-80 minutes).

Many respondents found the discussion of comprehensive liver cancer control a complicated task. As one respondent put it "*My gosh, that is a 40 hour discussion, it would cover many things*", and another cautioned at the end of a detailed discussion "*Those are some points *[but] *I am not being complete*." An example of the range of problems that need to be addressed by a comprehensive liver cancer control program was conveyed by one respondent:

"We will need to start with identifying the patients at-risk, we would then, after identifying those patients, need to come out with a surveillance strategy to monitor these patients regularly to minimize the chance that we overlooked the development of liver cancer in these patients, then we will need to have a general guideline on who should treat these patients meaning that they should be treated in specialized liver cancer centers that should be part of comprehensive cancer centers, and then we would need to have a study program using new drugs for the adjuvant treatment of those patients that have been treated and also palliative strategies to provide the best level of care for patients with incurable liver cancer."

Based on these interviews, 11 possible strategies of a comprehensive liver cancer control plan emerged as key themes, including *Access to treatments; Centers of excellence; Clinical education; Measuring social burden; Monitoring of at-risk populations; Multidisciplinary management; National guidelines; Public awareness; Research infrastructure; Risk-assessment and referral*; and *Transplantation infrastructure*. Rather than focus on the presentation of key quotes, we analyzed the data and worked with content experts (MK, KO, KH and SY) to elaborate a description of each of the 11 strategies (see table 1). This ensured that the findings constituted both a grounded and coherent interpretation of the data.

**Table 1:** Strategies for comprehensive liver cancer control

Strategy	Description	Relevant quotes
Access to treatments	Appropriate coverage and reimbursement for necessary prevention, surveillance, treatment, pain relief and palliative services.	*"Creating access to treatment-screening is a waste of effort if you don't link it to care"* *"The national insurance system does not fully cover payment"* *"Patients ask for new treatments, however, they are not covered by insurance"* *"It is important to eradicate drug lag and make good medication available as soon as possible"*

Centers of excellence	Specialized liver cancer centers to provide coordinated surveillance, treatment and research within a national liver cancer program.	*"Transfer patients with a HCC diagnosis to a tertiary hospital to receive state-of-the-art treatment"* *"There is no organization that brings all liver cancer research together under one roof"* *"Build a large center, experienced with international techniques, with a large number of patients"* *"We need to continue to preach to establish centers of excellence with multidisciplinary efforts"*

Clinical education	Improve primary care provider's awareness of the benefits of screening and early treatment, and necessary skills in risk assessment.	*"Most of the educational resources need to go into educating healthcare professionals"* *"Increase awareness among general practitioner, most are not aware"* *"Education of general practitioners concerning the screening of HCC, and gastroenterologists too"* *"We need to focus on the general education for primary care physicians so they will become vigilant"*

Measuring social burden	Accurate measures of risk factors, cirrhosis, liver cancer, the societal costs of illness and the benefits of improving liver cancer care.	*"Research the epidemiology of liver cancer, I think that we underestimate liver cancer by 50%"* *"Prevalence, surveillance, burden of disease, effective and cost-effective strategies"* *"Know the epidemiological trend for non-alcohol fatty liver disease and its impact on HCC incidence"* *"We need to have some comparison about how many lives we can save if we improve"*

Monitoring of at-risk populations	National surveillance programs for at-risk patients through expert services to diagnose HCC in early stages and improve outcomes.	*"Get at-risk patients into adequate screening programs at appropriate intervals and tested by experts"* *"Of cause surveillance programs are important to prevent or to detect early HCC"* *"There should be a national surveillance program for liver cirrhosis"* *"Monitor high-risk patients so if they develop HCC they can be diagnosed at an early stage and treated"*

Multidisciplinary management	Diagnosis, treatment decisions and follow-up of all HCC patients through collaborative teams of all relevant specialists.	*"Follow-up of HCC patients should be in a multidisciplinary team of different specialists"* *"Collaboration among physicians, surgeons, radiologists and oncologists is very poor"* *"Create an appropriate interdisciplinary board where every single patient is evaluated by this team"* *"It is very important to appreciate that this disease is heterogeneous with regards to the etiology"*

National guidelines	National standards for diagnosis and guidelines for screening, surveillance, treatment and palliation related to liver cancer.	*"There are no national guidelines on how to deal with patients with liver cancer"* *"There should be a national treatment strategy recognized and outcomes captured"* *"There is a lack of standardization of clinical diagnosis and treatment"* *"Information exchange among world leaders to prepare a global standard for prevention and treatment"*

Public awareness	Programs to improve public/political awareness about risk factors, surveillance, and survival benefits, and organized patient advocacy.	*"Greater public awareness of liver disease, risk factors and the fact that good treatments are available"* *"There is an absolute ignorance among the public and there is a clear need for education"* *"Patient groups are limited to popular types of cancer, but HCC is mainly the cancer of the poor"* *"Support experts to handle the details of patient advocacy so prevention and treatment could benefit"*

Research infrastructure	Funding, personnel, and facilities to conduct relevant basic, clinical and translational liver cancer research throughout the health system.	*"There is no specific program for HCC with public funding ... research infrastructure is always needed"* *"Train physicians who can lead clinical trials ... we also need research nurses"* *"Get thorough scientific research for HCC, genetics, biology, the pathways, it is very important"* *"There is an uneven distribution of research funding and the lack of grass-roots research funding"*

Risk-assessment and referral	Risk stratification conducted by primary care providers who refer patients to appropriate surveillance provided regularly by experts.	*"Identify at-risk patients, encourage them to be screened, and link them to appropriate care"* *"Primary doctors should not be treating viral hepatitis, they should be detecting it"* *"GPs don't consider it necessary and don't perform screening in patients with diagnosed cirrhosis"* *"We have very inefficient tools for identifying the high risk patients"*

Transplantation infrastructure	Improve awareness and capacity for organ donation, more capacity for transplantation, and alternatives to cadaveric transplantation	*"The situation cannot be altered without donors, but there is not much social infrastructure to support it"* *"It has been a major necessity to promote more cadaveric liver transplantation for more than decade"* *"The only shortcoming is transplantation, cadaveric transplantation is standard in other countries"* *"Real awareness of organ donation. There are some examples in the media, but still nothing happens"*.

Representative quotes remain unidentified to ensure anonymity and confidentiality of respondents

### Prioritization (qualitative)

Initial prioritization was based on the frequency with which the 11 strategies were discussed by the 20 respondents (but not accounting for multiple references within a single interview). The frequency and rank ordering of priorities are presented in table 2. The frequency of discussion across the key themes varied between 20-85%.

**Table 2:** Importance of strategies for liver cancer control

	Qualitative	Quantitative			
	
Strategy	Frequency (Rank)	OLS (SE)	P-Value (Rank)	Logit (SE)	P-Value (Rank)
Access to treatments	70%	0.192	0.010	1.146	0.004
	(5)	(0.07)	(3)	0.40	(2)

Centers of excellence	25%	0.192	0.001	1.079	0.002
	(10)	(0.05)	(2)	0.35	(3)

Clinical education	65%	0.108	0.055	0.625	0.019
	(6)	(0.05)	(8)	0.27	(7)

Measuring social burden	30%	-0.025	0.698	-0.185	0.613
	(8)	(0.06)	(11)	0.37	(10)

Monitoring of at-risk populations	80%	0.275	< .001	1.508	< 0.001
	(2)	(0.06)	(1)	0.41	(1)

Multidisciplinary management	35%	0.158	0.004	0.919	0.004
	(7)	(0.05)	(4)	0.32	(4)

National guidelines	80%	0.158	0.025	0.678	0.056
	(2)	(0.06)	(6)	0.36	(6)

Public awareness	75%	0.158	0.007	0.841	0.018
	(4)	(0.05)	(5)	0.36	(5)

Research infrastructure	20%	0.058	0.243	0.337	0.178
	(11)	(0.05)	(9)	0.25	(9)

Risk-assessment and referral	85%	-0.008	0.870	-0.135	0.645
	(1)	(0.05)	(10)	0.29	(11)

Transplantation infrastructure	30%	0.125	0.196	0.352	0.543
	(8)	(0.09)	(7)	0.58	(8)

Robust standard errors in parentheses.

The most discussed items were *Risk-assessment and referral *(85%), *National guidelines *(80%) and *Monitoring of at-risk populations *(80%) implying that they are potential priorities. *Research infrastructure *(20%), *Centers of excellence *(25%), *Measuring social burden *and *Transplantation infrastructure *(both 30%) were strategies that were discussed with the lowest frequency, implying a lower priority.

### Prioritization (quantitative)

Invitations were sent to 42 potential respondents. Of these 23 (55%) consented to participate and 20 were eligible to participate. Field workers noted that after the first interviews in each country, which were all supervised by a senior investigator (JB or BB), respondents reported some difficulty with the choice tasks, mainly due to a lack of familiarity with conjoint analysis methods. Based on these concerns, all field workers discussed these difficulties, and strategies to overcome this problem were discussed. Here an example question, that was completed and explained, was added to ensure that all respondents were comfortable with the survey instrument and that all respondents were managed in a way that was consistent with these early interviews. This resolved the issue, with the remaining responders reporting no difficultly with the tasks.

Table 2 presents the importance weights (i.e. parameter estimates) from choice models estimated from the conjoint analysis data using both a linear probability model (estimated via ordinary least squares) and logistic regression. Robust standards errors, p-values (based on a two tailed test) and rankings of priorities are also shown. Statistical significance (p < 0.05) was achieved on six strategies for both methods, with both methods in agreement on the significance on the top five factors. Here *National statistics *was significant based on OLS (p = 0.025), but not based on the logistic model (p = 0.056). Likewise, *Clinical education *was significant when considering logistic estimation (p = 0.019), but not when using OLS (p = 0.055). Both methods identified *Measuring social burden *and *Risk-assessment and referral *as having negative importance weights, but neither aversion reach statistical significance. Overall, there was a very-high level of agreement between the two methods (rho = 0.979; p < 0.0001), so substantive findings are drawn only from the logistic estimation.

As seen in table 2 the highest priority as estimated using the conjoint analysis was *Monitoring of at-risk populations *(p < 0.001), followed by *Access to treatment *(p = 0.004), *Centers of excellence *(p = 0.002), *Multidisciplinary management *(p = 0.004), *Public awareness *(p = 0.018), *National guidelines *(p = 0.056) and *Clinical education *(p = 0.019).

### Comparison of qualitative and quantitative priorities

**Figure 2:**
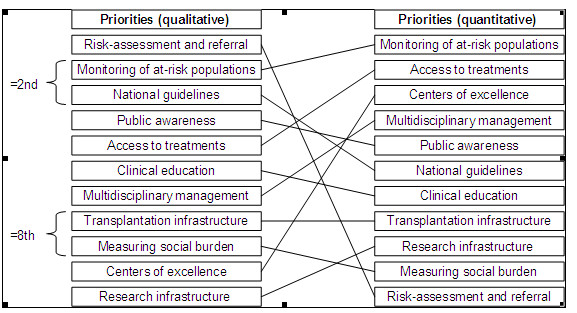
**A comparison of priorities using qualitative and quantitative methods**.

When comparing the priorities from the conjoint analysis to the frequency analysis based on the qualitative data, there was some positive correlation (rho = 0.20), but this relationship was not significant (p = 0.554). When considering the priority given to individual attributes (see Figure 2), similar importance (as indicated by their rank) was given to *Monitoring of at-risk populations *(qual = 2/quant = 1), *Public awareness *(qual = 4/quant = 5), *Access to treatment *(qual = 5/quant = 2), *Clinical education *(qual = 6/quant = 7), *Multidisciplinary management *(qual = 7/quant = 4), *Transplantation infrastructure *(qual = 8/quant = 8), *Measuring social burden *(qual = 8/quant = 10), and *Research infrastructure *(qual = 11/quant = 9). Differences in priority between the two methods were found for *Risk-assessment and referral *(qual = 1/quant = 11) and *Centers of excellence *(qual = 10/quant = 2), and to a lesser extent *National guidelines *(qual = 2/quant = 6).

## Discussion

**Figure 3:**
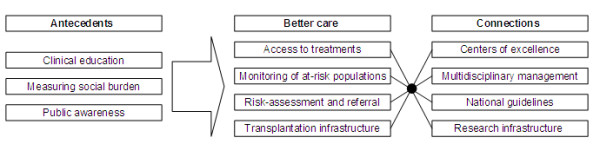
**The ABCs of comprehensive live cancer control**.

When one considers the 11 strategies for comprehensive liver cancer control identified in this paper, we can see that they cover factors associated with facilitating, providing and integrating care into a single system. To facilitate the possible implementation of these strategies, one can conceptualize them into three categories: antecedents; better care; and connection. As seen in Figure 3, this can lead to a model that relates to the ABCs of comprehensive liver cancer control. Here *Antecedents *include *Clinical education*, *Measuring social burden *and *Public Awareness*, all factors that can motivate the adoption of comprehensive liver cancer control. Access to treatments, Monitoring of at-risk populations, Risk-assessment and referral and Transplantation infrastructure are all factors aimed at providing *Better care*, a vital component of any comprehensive cancer control plan. Finally, a well functioning system must have its components well connected. In our model, *Centers of excellence*, *Multidisciplinary management*, *National guidelines *and *Research infrastructure *are important *Connections *of a comprehensive liver cancer control plan.

The strategies identified here parallel some of the strategies embedded in the WHO guidelines for general comprehensive cancer control with two exceptions [9]. First, strategies for pain relief/palliative care were not identified as an important cancer control strategy by our clinical respondents. This may have been different if more variety in the types of stakeholders were included in our sample (e.g. we had no nurses, patients or advocates), but there may be a lack of advocacy for liver cancer more generally [54]. This said, pain relief/palliative care can be seen as belonging to our *Access to treatment *strategy. Second, we do not differentiate strategies for implementation in low, middle and high income countries [10], nor did we examine such heterogeneity in priorities. These two differences highlight the need for further research to differentiate priorities across different stakeholders (including advocates, where they may exist) and across different countries in Asia and beyond.

While this paper identified certain priorities for implementation in an Asian comprehensive liver cancer control plan, it is important to compare these to current policies in Asia. Highest priority was given to *Monitoring of at-risk populations*, which has been shown to facilitate early diagnosis [55]. Such surveillance programs are related to surveillance in primary care [56], which may account for the low value given to Risk-assessment and referral (i.e. respondents found the former more beneficial to the latter). This said, risk stratification may be important to target surveillance strategies [57]. While *Centers of excellence *were only discussed by a minority of respondents in the qualitative interviews, this strategy was considered a priority in the conjoint analysis. Such specialized centers have been shown to be of value in early surveillance and improved outcomes for HCC in Japan [58].

Priority was also placed on *Access to treatment *by respondents in both the qualitative and quantitative portions of this study. While lack of robust financing systems is a major barrier in many Asian countries, barriers to access persist in those countries with national health insurance. Other barriers include a lack of reimbursement, high copayments, a lack of specialty centers, the availability of specialists and awareness of the disease among primary care physicians and the general public [59]. A shortage of organ donors and subsequent waiting lists also pose barriers to access to transplantation [60].

There are some omissions from our set of strategies. For example, in addition to the absence of pain management and palliative care, our study did not specifically characterize hepatitis control as a strategy. However, prevention (e.g. through HBV vaccination), treatment and control (which would include treatment of hepatitis associated with HCC) are within the descriptors for *Access to treatment*. While quality hepatitis control exists in many Asian countries [61,62], hepatitis control must be a priority in many countries not included in this study [63,64].

It is also important to consider some alternative interpretations of the data in this study. One interpretation is that valuation of some strategies in the qualitative analysis may be as a result of framing effects in the presentation of the choice tasks. For example, *Measuring social burden*, which received a negative value, was described as "Measuring the social burden of liver cancer" (see Figure 1). Here respondents may have found this label ambiguous, or as implying factors that were not implicit in the qualitative analysis. This label may have been better described with terms such as measuring incidence or prevalence, terms that are more familiar to the respondents.

*Risk-assessment and referral *received a negative valuation despite being the most frequently discussed strategy in the qualitative analysis. Here several factors may have contributed to this aversion. First, the factor was described in the conjoint tasks as "Improved risk-assessment and referral by primary care" (see Figure 1), and the "improved" may have unduly framed the factor (especially for countries that have good risk-assessment mechanisms) or made the factor ambiguous (especially for those who do not have such mechanisms). Second, it may have been more accurate to refer to this as "continuous surveillance" of at risk populations. Third, there was some confusion between "risk-assessment and referral"-mechanisms to stratify those at-risk of developing HCC and referring them to appropriate care-and "monitoring of at-risk populations"-mechanisms of surveillance for patients identified as being at-risk, preferably in specialty care. Finally, there may be heterogeneity in the valuation of this factor across the study countries, i.e. this may be a priority in some countries, but not in others, potentially because such services are already provided or because systems are not based upon primary care providers originating risk assessment and diagnosis.

While this study is motivated by a need for comprehensive liver cancer control in Asia [10], it also highlights a more general need for more quantitative research methods to guide priority setting in health care. The prioritization of limited resources across competing demands presents an "*economic challenge and a political puzzle" *[65], but is a vital element of systematic planning in public health [66]. While some health care planners utilize multiple evidence sources (both qualitative and quantitative) for the purposes of priority setting [67], stakeholder engagement in policy often is limited to nominal groups or consensus-based approaches (e.g. Delphi techniques) [68,69]. As such, this study makes a significant contribution towards demonstrating the value of conjoint analysis in prioritization of health care policy strategies and challenges the soundness of consensus-based approaches.

## Conclusions

Identified strategies can be conceptualized as the ABCs of comprehensive liver cancer control as they focus on *Antecedents*, *Better care *and *Connections *within a national strategy. Some concordance was found between the qualitative and quantitative methods (e.g. *Monitoring of at-risk populations*), but substantial differences were also identified (e.g. qualitative methods gave highest priority to risk-assessment and referral, but it was the lowest for the quantitative methods), which may be attributed to differences between the methods and study populations, and potential framing effects in choice tasks. Continued research will provide more generalizable estimates of priorities and account for variation across stakeholders and countries.

## Competing interests

The authors declare that they have no competing interests.

## Authors' contributions

JB and BB conceptualized the study, designed the study instrument and made substantial contributions to the data interpretation and writing of the paper; MK, KO, K-HH and S-LY participated in the instrument design, recruitment and interpretation of the data; GG participated in data analysis and drafting of the final manuscript. All authors read and approved the final manuscript.

## Funding

This study was funded, in part, by Bristol-Myers Squibb. The funders had no role in study design, data collection and analysis, selection of respondents, decision to publish, or preparation of the manuscript.

## Pre-publication history

The pre-publication history for this paper can be accessed here:

http://www.biomedcentral.com/1472-6963/11/298/prepub
